# Case report: From palliative to potentially curative – the advent of immunotherapy providing hope to advanced gallbladder adenocarcinoma

**DOI:** 10.3389/fimmu.2024.1353430

**Published:** 2024-02-02

**Authors:** Eugene Kwong Fei Leong, Nigel Chun Hian Tan, Ning Qi Pang, Alfred Wei Chieh Kow

**Affiliations:** ^1^ Division of Hepatobiliary & Pancreatic Surgery, Department of Surgery, National University Hospital, Singapore, Singapore; ^2^ Yong Loo Lin School of Medicine, National University of Singapore, Singapore, Singapore; ^3^ Division of Surgical Oncology, National University Cancer Institute, Singapore, Singapore

**Keywords:** gallbladder cancer, biliary tract cancer, peritoneal metastasis, immunotherapy, conversion surgery, hepatectomy, case description

## Abstract

**Introduction:**

Biliary tract cancers (BTC) are often diagnosed at an advanced stage where prognosis is poor and curative-intent surgery is infeasible. First-line cisplatin-gemcitabine chemotherapy for advanced gallbladder cancer has remained unchanged over more than a decade, but recent developments in immunotherapy such as durvalumab have highlighted promise as a combination treatment regime with current standard chemotherapy.

**Methods:**

In this case description, we present a case of locally-advanced gallbladder adenocarcinoma involving the biliary confluence that was initially planned for an extended right hepatectomy after portal vein embolization. Interval imaging revealed peritoneal metastasis, which was confirmed on diagnostic laparoscopy and biopsy. The patient underwent 8 cycles of cisplatin 25 mg/m2 and gemcitabine 1,000 mg/m2 chemotherapy on days 1 and 8 of each 21-day cycle, with durvalumab (Imfinzi^®^) 1,500 mg immunotherapy on day 1 of every cycle, in accordance with the treatment protocol of the TOPAZ-1 trial. Repeat imaging demonstrated a stable primary lesion with no further evidence of peritoneal disease. The patient subsequently underwent curative-intent conversion surgery with an extended right hepatectomy and Roux-en-Y hepaticojejunostomy, which were completed through a fully minimally-invasive laparoscopic approach.

**Results:**

Final pathological TNM classification was ypT1aN0, with near-complete pathological response to pre-surgical therapy, uninvolved margins (R0 resection) and tumour shrinkage from 2.5 centimetres on pre-operative cross-sectional imaging to 0.5 centimetres on final histology. The patient had an uneventful post-operative course, and was fit for discharge by the fourth post-operative day. He remained well after three months of routine post-operative follow-up, with no significant post-operative complications and biochemical or radiological evidence of disease recurrence.

**Conclusion:**

Our case description highlights the immense potential of combination durvalumab immunotherapy with cisplatin-gemcitabine chemotherapy in the treatment of advanced gallbladder adenocarcinoma. The patient’s locally advanced disease was initially planned for complex open surgery, prior to discovery of peritoneal metastasis rendering it inoperable. This was successfully down-staged with combination therapy to eventual R0 resection via minimally-invasive surgery. In addition, this case description demonstrates the feasibility of a fully laparoscopic approach with postulated benefits of diagnostic re-evaluation of peritoneal disease, reduced wound pain and shorter length of hospital stay.

## Introduction

Biliary tract cancers (BTCs) consist of a heterogenous group of epithelial cancers of the biliary tree, which include gallbladder cancer, ampulla of Vater cancer and cholangiocarcinoma (1). While the incidence of gallbladder cancer is low, diagnosis is often at an advanced stage (1–3). For the subset of patients deemed to have resectable disease, surgery is the cornerstone of curative therapy and approach is dependent on the location of the cancer (1). For unresectable or metastatic disease, prognosis is poor, curative surgery is infeasible and management is palliative (1–3). Standard first-line cisplatin-gemcitabine combination chemotherapy has remained unchanged for the past decade since the UK ABC-02 trial, which demonstrated median overall survival (OS) of 11.7 months and progression-free survival (PFS) of 8.0 months (4).

There has been an ongoing search for additional systemic strategies for patients with advanced gallbladder cancer, including triplet cytotoxic combinations, targeted therapy and immunotherapy (2). In 2022, the phase 3 TOPAZ-1 trial evaluated the addition of durvalumab immunotherapy to standard cisplatin-gemcitabine chemotherapy compared to placebo for treatment-naïve advanced BTC and demonstrated improvement in median OS from 11.5 months to 12.8 months, with improvement in objective response rate (ORR) from 18.7% to 26.7% (5).

To the authors’ knowledge, there are no detailed reports of fully minimally-invasive surgical resection and reconstruction following successful downstaging of advanced gallbladder adenocarcinoma with peritoneal metastasis with combination durvalumab immunotherapy with cisplatin-gemcitabine chemotherapy. We herein present our experience and suggest feasibility of this approach with the evolving landscape of systemic therapy for advanced BTC.

## Case description

**Figure 1:**
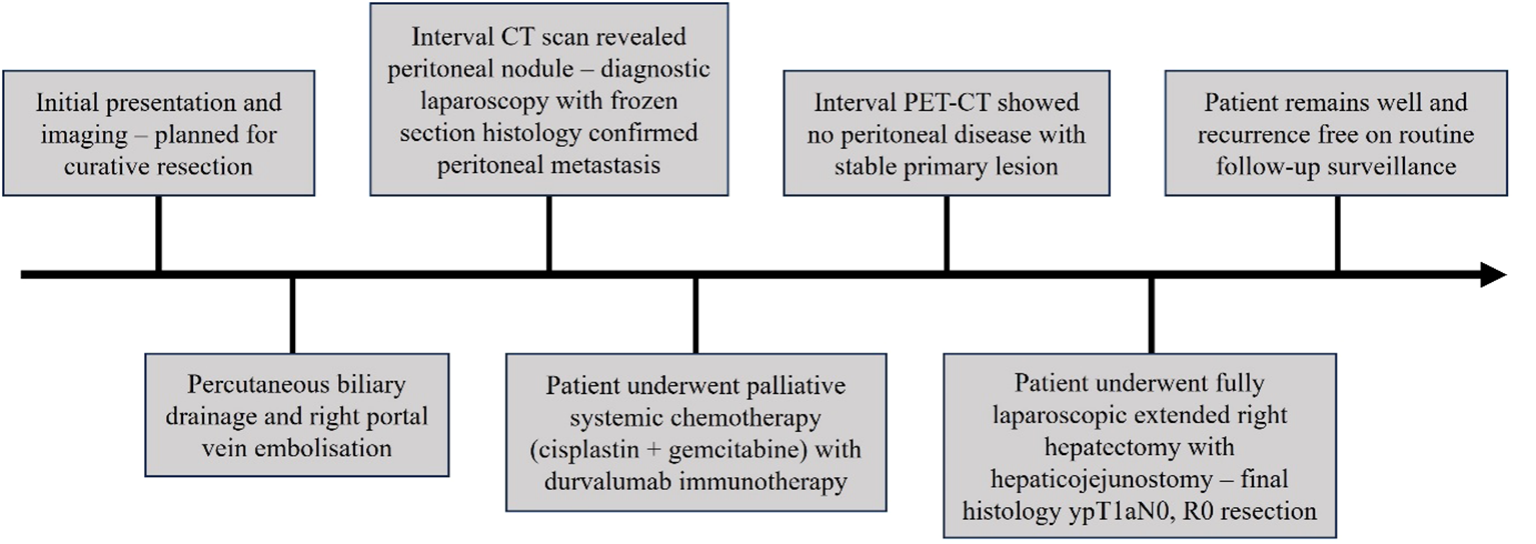
Timeline of patient’s treatment.

**Figure 2:**
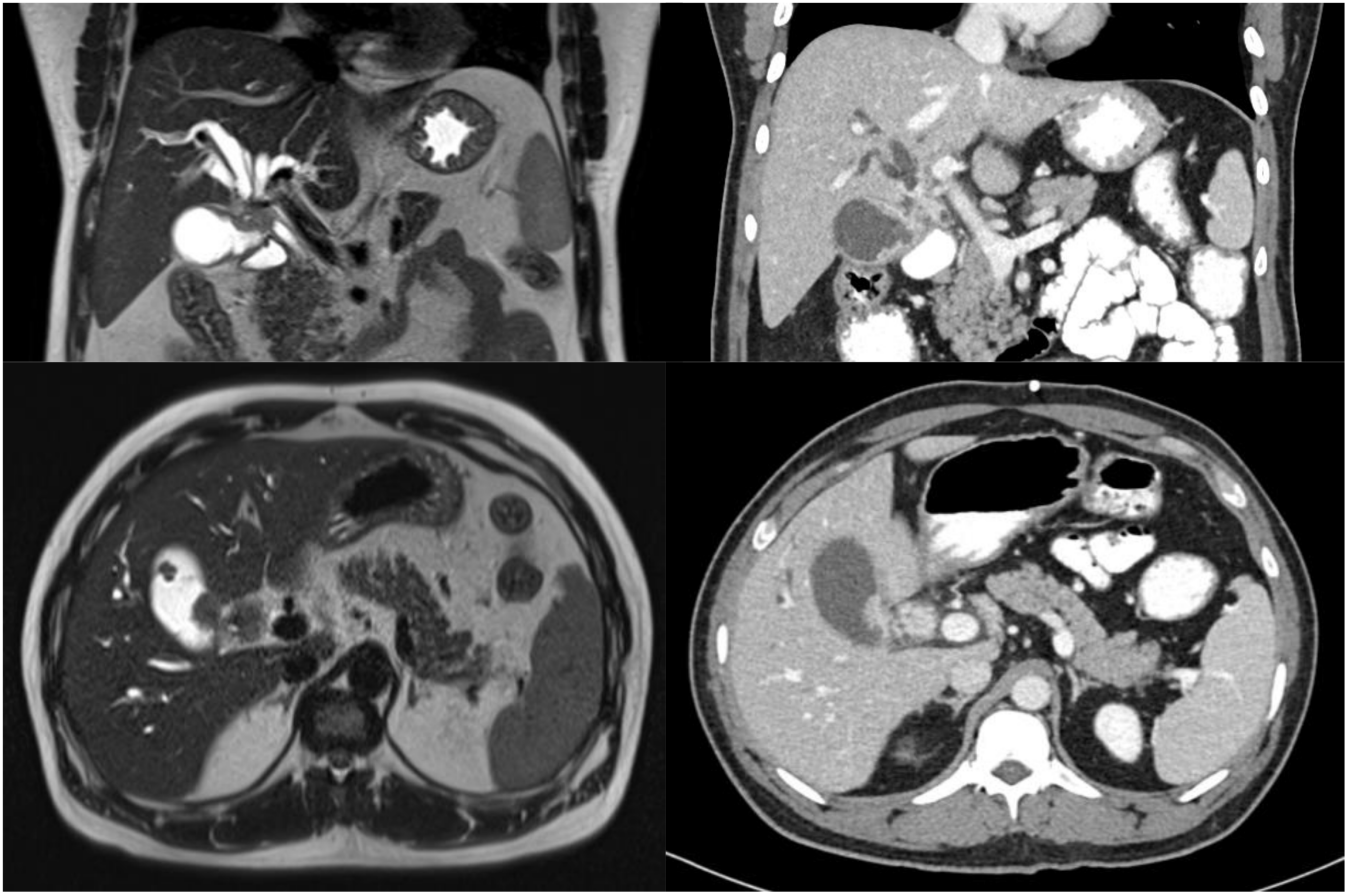
Initial MRI (left) and CT (right) scans demonstrating soft tissue mass involving the common hepatic duct bifurcation, cystic duct and gallbladder.

Our patient was a 39-year-old gentleman with no significant past medical history or familial history of malignancy. He initially presented with right hypochondrium pain and jaundice (**Figure 1**). Abdominal examination did not yield any palpable masses or tenderness. Liver function tests revealed conjugated hyperbilirubinaemia with total bilirubin 200.1 μmol/L (normal range 3.4-25.6 μmol/L) and direct bilirubin 147.1 μmol/L (normal range 0.0-8.6 μmol/L), and a hepatocellular pattern of transaminitis. Serum CA 19-9 was 46.7 U/ml (normal range 0.0-39.0 U/ml). The patient had a body mass index of 29.0 kilogrammes per square metre, was a social smoker and did not have known history of gallstone disease or polyps. Gadobutrol-enhanced magnetic resonance imaging (MRI) and contrast-enhanced computed tomography (CT) scans of the abdomen revealed a 2.5 by 1.6-centimetre Bismuth-Corlette type 3B irregular infiltrative soft tissue thickening involving the common hepatic duct bifurcation and left hepatic ducts, with resultant bilateral intrahepatic bile duct dilatation (**Figure 2**). This mass extended to involve the adjacent cystic duct, as well as the neck and proximal body of the gallbladder, measuring 1.0-centimetre in maximal thickness within the proximal body. In addition, there were internal septations and numerous lobulated polypoid lesions in the gallbladder – the largest of which measuring 2.2-centimetres. Both the bile duct and gallbladder lesions demonstrated low signal on T2-weighted sequences, and mildly low signal with enhancement on T1-weighted sequences. There was a 1.3-centimetre peri-portal lymph node but no obvious distant metastasis. The preliminary diagnosis was either infiltrative gallbladder cancer involving the hepatic duct confluence or concurrent primary hilar cholangiocarcinoma and gallbladder cancer.

**Figure 3:**
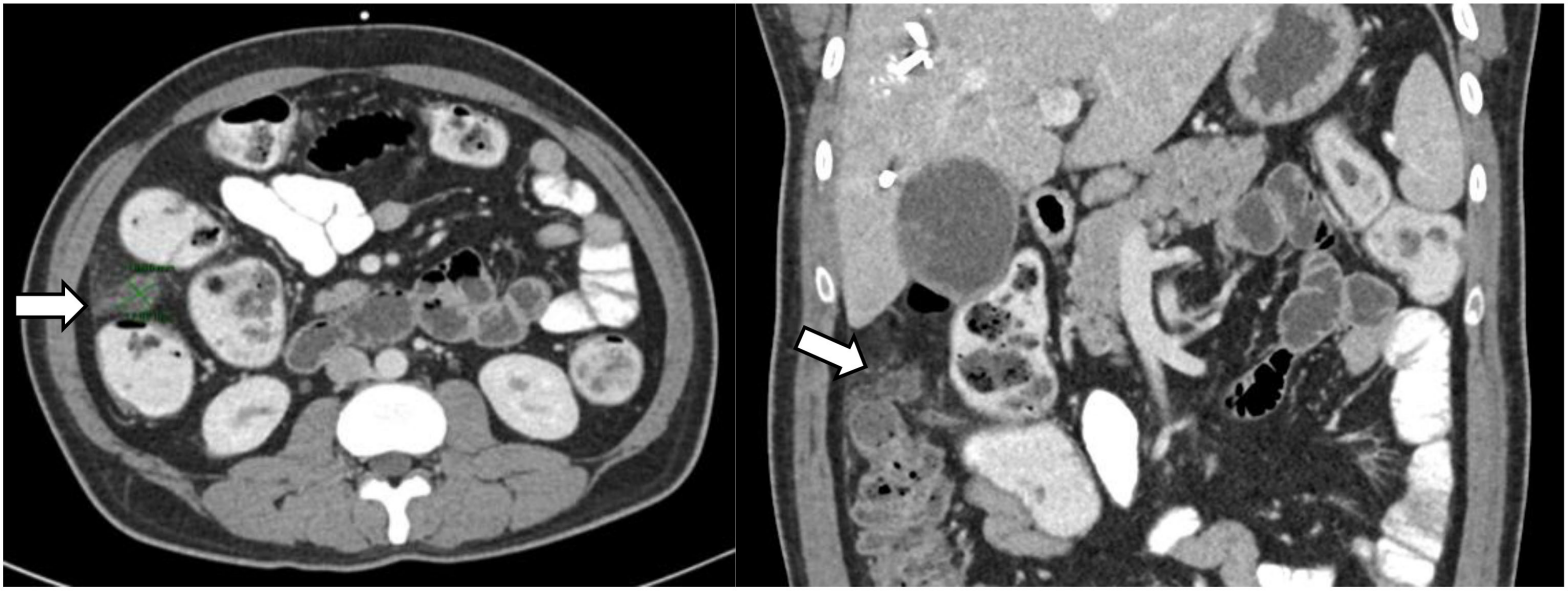
Interval CT scan after right portal vein embolisation demonstrating new 2-centimetre intra-peritoneal soft tissue lesion (white arrow), suspicious for peritoneal metastasis—this was eventually confirmed on diagnostic laparoscopy and biopsy.

The patient was initially deemed suitable for curative-intent surgery, and was planned for an extended right hepatectomy with caudate lobe and bile duct resection. He underwent percutaneous transhepatic biliary drainage and right portal vein embolization to allow compensatory left liver hypertrophy, as the future liver remnant was small at initial volumetric assessment. An interval abdominal computed tomography scan with volumetry two months later demonstrated tumour progression of the hilar lesion to involve the proximal left hepatic duct. Furthermore, this scan revealed small volume ascites and an enhancing 2.0 by 1.8-centimetre soft tissue lesion at the right subhepatic region with reticulation of the adjacent omentum, which was radiologically suspicious for peritoneal metastasis (**Figure 3**). Diagnostic laparoscopy demonstrated extensive peritoneal disease, with numerous nodules on the coronary and falciform ligaments, as well as in the right subphrenic, left lower abdomen and suprapubic regions. On-table frozen section histopathology confirmed metastatic moderately-differentiated adenocarcinoma. The patient underwent radiologically-guided insertion of a 6-centimetre self-expandable metallic stent across the biliary obstruction with no local anti-tumoral therapy, and was referred to a medical oncologist for palliative systemic therapy.

The patient underwent 8 cycles of cisplatin 25 mg/m^2^ and gemcitabine 1,000 mg/m^2^ chemotherapy on days 1 and 8 of each 21-day cycle, with durvalumab (Imfinzi^®^) 1,500 mg immunotherapy on day 1 of every cycle, in accordance with the treatment protocol of the TOPAZ-1 trial (5). Thereafter, an interval positron-emission tomography and computed tomography (PET-CT) scan confirmed a stable primary lesion with no further evidence of peritoneal disease. Vascular anatomy of the left hepatic lobe was not radiologically involved by tumour invasion and was consistent with prevalent anatomy. Serum CA 19-9 levels fell to normal ranges of 28.2 U/ml at 8 weeks after completion of systemic treatment, and 4.0 U/ml at 10 weeks. In view of the patient’s young age and favourable radiological and biochemical response to systemic therapy, he was offered another attempt at curative-intent resection. The expected volume of the future liver remnant was 683 millilitres, or 52.8% of the estimated standard liver volume.

**Figure 4:**
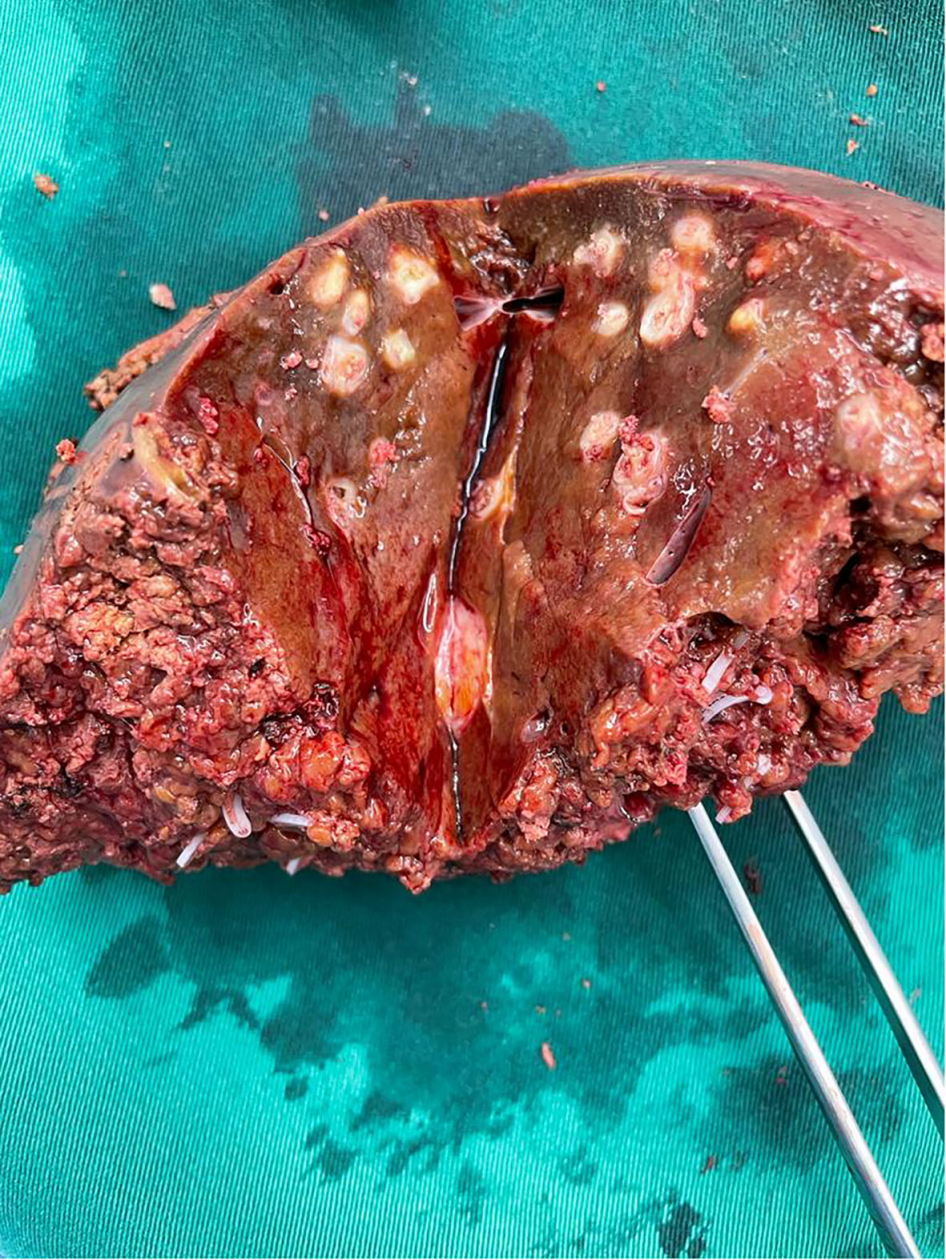
Clinical photograph of bisected specimen showing post-embolisation changes – there were no liver metastases on gross pathology or on microscopic examination.

At diagnostic laparoscopy, a thorough examination of the peritoneal surfaces yielded no obvious metastatic disease or ascites. Extended right hepatectomy and reconstruction via a left hepaticojejunostomy was completed via a fully laparoscopic approach, with parenchymal dissection using a CUSA^®^ device (Integra LifeSciences, New Jersey, United States). An end-to-side roux-en-Y hepatico-jejunostomy was performed laparoscopically with left hepatic duct anastomosis to jejunum using Stratafix™ PDS 4-0 (Ethicon, New Jersey, United States) in a single-layer continuous technique. Specimen extraction was via the umbilical incision (**Figure 4**).

The patient had an uneventful recovery and was fit for discharge by the fourth post-operative day. Final histology revealed moderately-differentiated gallbladder adenocarcinoma with few foci of viable tumour invading the lamina propria. 2 lymph nodes were negative for malignancy, and resection margins were uninvolved. There was near-complete pathological response to pre-surgical therapy, and eventual tumour size was 0.5 centimetres, from an initial size of 2.5 centimetres. Final TNM classification (8^th^ American Joint Committee on Cancer, AJCC) was ypT1aN0. The patient remained well after six months of routine post-operative follow-up, with no significant post-operative complications or evidence of disease recurrence. Bilirubin and transaminases were normal, with mildly elevated serum alkaline phosphatase of 134 U/L. Post-operative CA 19-9 was not elevated, at 10.8 U/ml and remained normal at follow-up 6 months after surgery. The patient’s case was rediscussed at the tumour board and recommended for close post-operative surveillance and adjuvant therapy. He received a further 4 cycles of gemcitabine, cisplastin and durvalumab in 21-day cycles, followed by maintenance durvalumab,

Informed consent was obtained from the patient for publication as a case description.

## Discussion

Outcomes in advanced and unresectable BTC remain poor and an ongoing unmet need. The current standard of care consists mainly of regimens such as cisplatin-gemcitabine with or without the addition of nab-paclitaxel or S-1 (2, 6, 7). Research into novel therapeutic approaches for BTC have found unique targets such as isocitrate dehydrogenase 1 (IDH1), human epidermal growth factor 2 (HER2) and BRAF V600E mutations, as well as fibroblast growth factor receptor 2 (FGFR2) and neutrotrophic tyroskine kinase (NTRK) fusions (8). Several phase 1 and 2 trials are underway—however, current clinical utility appears limited by the low prevalence of the respective targetable mutations in BTCs (2).

As with several other cancers like melanoma and non-small cell lung cancer, the recent introduction of immune checkpoint inhibitors (ICIs) has significantly changed the landscape of systemic therapy for BTC (2, 9). Cancers evade anti-tumour immune responses through various immune escape mechanisms, including local tumour microenvironment modulation with establishment of an immunosuppressive milieu, loss of MHC expression, as well as expression of programmed cell death protein 1 (PD-1) and cytotoxic T-lymphocyte-associated antigen 4 (CTLA-4) (10). Evidence suggests that BTCs are immunogenic cancers — 46-94% of BTC express PD-L1, and this is found to be associated with poorer survival outcomes (11–13).

However, only a small proportion of patients with BTC have predictors of response to immunotherapy, such as mismatch repair deficiency, high microsatellite instability (MSI) or high tumour mutational burden (14–17). Furthermore, early trials have seen limited activity in monotherapy, but sustained responses in the minority of patients responding to treatment (1, 18). The KEYNOTE-28 phase 1b trial studied the efficacy of the PD1 monoclonal antibody pembrolizumab in various types of cancers, including 24 patients with programmed death ligand 1 (PD-L1) positive BTC that had progressed despite standard treatment – PD-L1 positivity was defined as ≥1% staining of tumour nest cells or IHC assay presence of PD-L1 positive bands in stroma. Overall response rate (ORR) was 13.0% (all partial response), median OS was 5.7 months and median PFS was 1.8 months (18, 19). The KEYNOTE-158 phase 2 trial evaluated the efficacy of pembrolizumab monotherapy in several advanced solid tumours including 104 BTC patients, regardless of biomarker status. ORR was 5.8% (all partial response), median OS was 7.5 months and median PFS was 2.0 months (18). More recently, the multi-national KEYNOTE-966 phase 3 trial demonstrated improved overall survival with addition of pembrolizumab to gemcitabine and cisplatin chemotherapy in 1069 patients with unresectable locally-advanced or metastatic BTC, at 12.7 months with pembrolizumab compared to 10.9 months without (20).

In 2022, results of the 685-patient TOPAZ-1 study sought to redefine the first-line standard of care treatment for previously untreated advanced BTC. This double-blind, placebo-controlled, international phase 3 study concluded that addition of durvalumab immunotherapy to standard cisplatin-gemcitabine therapy improved median OS from 11.5 months to 12.8 months, and 24-month OS from 10.4% to 24.9%. The ORR, a summation of complete and partial responses, was 18.7% in the placebo group and 26.7% in the durvalumab group (5). This benefit was regardless of PD-L1 expression, and was observed in all population subtypes (5, 21). About half of the patients in each treatment group did not have MSI status, but MSI-high tumours were seen in 1.5% of those evaluated for MSI status (5). The results of this study highlighted promise for immunotherapy in advanced BTC, and this was reflected in the recent FDA approval of durvalumab plus standard chemotherapy as first-line treatment in such patients (2, 22).

Limited information is available about the outcomes of surgical resection after immunotherapy in advanced BTC, but existing studies report various immunotherapy agents (pembrolizumab, tislelizumab, sintilimab, camrelizumab, toripalimab, ipilimumab or nivolumab) alone, or in combination with chemotherapy (cisplatin-gemcitabine or oxaliplatin-gemcitabine) or with lenvatinib, a multiple kinase inhibitor (23–26).

In an open-label, single-centre, prospective phase 2 trial by Zhang et al. in 2021, 38 patients with initially unresectable or metastatic BTC received lenvatinib once daily (8mg if body weight <60kg, 12mg if body weight ≥60kg) in addition to an intravenous PD-1 inhibitor once every 3 weeks (pembrolizumab 200mg, tislelizumab 200mg, sintilimab 200mg, camrelizumab 200mg or toripalimab 240mg) (23). Study participants had not received prior chemotherapy. Overall median OS was 17.7 months, and 13 patients (34.2%) achieved adequate tumour response to be eligible for conversion surgery (23). In the group of patients undergoing surgery, 12 (92.3%) had an R0 resection, while 6 patients (46.2%) had post-operative complications including 2 cases of bile leak, 2 cases of pleural effusion, 1 case of gastrointestinal bleeding and 1 case of delayed recovery of liver function (23). There was no in-hospital post-operative mortality following surgery, and event-free survival (EFS) was 13.5 months (95%CI 13.0-14.0 months) compared to 4.6 months (95%CI 0.8-8.4 months) for patients who only received systemic therapy and no surgery (23). While this study is likely to represent the largest reported cohort of patients undergoing conversion surgery following immunotherapy, the study participants did not receive any chemotherapy prior to or during the study, which in combination with durvalumab immunotherapy is likely to form the new first-line treatment for advanced BTC.

In 2021, Satyananda et al. reported a case of cT3N1M0 (8^th^ AJCC) locally advanced gallbladder adenocarcinoma deemed to not be completely resectable at the time of initial diagnosis (24). After cisplatin-gemcitabine chemotherapy and subsequent ipilimumab and nivolumab immunotherapy, the patient experienced good radiological and tumour marker responses. He underwent right portal vein embolization followed by an open extended right hepatectomy, radical cholecystectomy and roux-en-Y hepaticojejunostomy reconstruction (24). Final histology demonstrated R0 resection with no positive lymph nodes and complete tumour response—eventual TNM classification was ypT0N0M0. The case patient was noted to be disease-free and well at 10-month follow-up (24).

2 patients from the CA209-538 study eventually underwent surgery for residual disease after achieving ongoing response with nivolumab and ipilimumab immunotherapy (25). This multi-centre, non-randomised, open-label, phase 2 trial in 2020 evaluated for disease control of three cohorts of rare cancers, including 39 patients with advanced BTC – of which 9 patients had an objective response (23%) (25). Nivolumab and ipilimumab were administered every 3 weeks for 4 doses during the induction phase, followed by nivolumab monotherapy every 2 weeks during the maintenance phase until disease progression, intolerable toxicity or upon reaching 2 years from the time of study enrolment. The authors noted that these 2 patients who subsequently had surgical resection following trial protocol were relapse-free at most recent follow-up, but did not elaborate further on sites of disease, surgical approach, operative outcomes, follow-up duration, OS and PFS (25).

Zhou et al. evaluated the use of toripalimab, lenvatinib, gemcitabine and oxaliplatin combination therapy in 30 patients with advanced intrahepatic cholangiocarcinoma in an open-label, single-arm, phase 2 trial in 2020 (26). This study reported an impressive ORR of 80% (24/30) with 1 patient achieving complete response. 2 patients in this study with locally advanced disease were successfully down-staged and underwent surgical resection (26). No additional detail was provided regarding the patients who subsequently underwent surgery.

This rapidly developing trend of successful downstaging from palliative-intent systemic treatment to potentially curative multi-modality therapy including oncological resection, provides hope to patients with advanced BTC – a diagnosis with a previously bleak prognosis. The patient in our case description was initially diagnosed to have BTC with metastatic peritoneal disease, and received cisplatin-gemcitabine chemotherapy in combination with durvalumab immunotherapy as per the TOPAZ-1 trial protocol. In view of his young age, excellent performance status and favourable PET imaging findings with no further peritoneal disease, he was offered an attempt at curative resection. The surgery was completed via a fully minimally-invasive approach for both tumour resection and hepatico-jejunostomy reconstruction, and the post-operative course was uneventful. Adequate pain control was easily achieved with minimal requirement of opioid analgesia, likely a result of the laparoscopic approach. Specimen histology confirmed R0 resection and near complete pathological response to pre-operative systemic treatment.

With the improvement of OS and ORR seen in the TOPAZ-1 trial and the resultant FDA approval for durvalumab immunotherapy to standard cisplatin-gemcitabine chemotherapy, it is expected that more patients will receive this treatment regime and present to the surgeon for consideration of conversation surgery. Our case description highlights the revolutionary value of TOPAZ-1 combination therapy, and the utility of a fully laparoscopic approach for R0 resection conversion surgery in several key benefits: i) combination cisplatin-gemcitabine chemotherapy with durvalumab immunotherapy successfully down-staged metastatic gallbladder adenocarcinoma from palliative-intent treatment to final curative-intent R0 surgical resection; ii) the utilisation of laparoscopy at the beginning of surgery allowing for a thorough evaluation of the peritoneal surfaces, which was essential in our patient due to previous diagnosis of peritoneal metastasis; ii) post-operative pain control could be easily achieved with an opioid-sparing regime of simple analgesia due to the minimally invasive approach; and iv) use of an Enhanced Recovery After Surgery (ERAS) care bundle resulting in a short hospital length of stay, with the patient deemed medically fit for discharge within four days after surgery. Further study into the long-term survival outcomes, disease-free survival, post-operative outcomes for conversion surgery and optimal surgical approach (ie laparoscopic versus open) for advanced BTC following durvalumab immunotherapy and cisplatin-gemcitabine chemotherapy is required.

## Conclusion

The cornerstone of systemic treatment of advanced BTC has been cisplatin-gemcitabine chemotherapy for more than a decade. The advent of novel systemic therapy options such as durvalumab immunotherapy has met promising results as demonstrated in the recent TOPAZ-1 trial, and may eventually form the new standard of care. Our case description highlights that patients with metastatic disease who respond favourably to systemic treatment can become candidates for potentially-resectable surgery. In our patient, this was successfully accomplished through a fully minimally-invasive approach for both oncological resection and bilio-enteric reconstruction.

## Data availability statement

The original contributions presented in the study are included in the article/**Supplementary Material**. Further inquiries can be directed to the corresponding author.

## Ethics statement

Ethical approval was not required for the studies involving humans because Case report with use of non-identifiable patient details. The studies were conducted in accordance with the local legislation and institutional requirements. The participants provided their written informed consent to participate in this study. Written informed consent was obtained from the individual(s) for the publication of any potentially identifiable images or data included in this article.

## Author contributions

EL: Conceptualization, Investigation, Methodology, Resources, Visualization, Writing – original draft, Writing – review & editing. NT: Investigation, Methodology, Resources, Visualization, Writing – review & editing. NP: Investigation, Methodology, Resources, Visualization, Writing – review & editing. AK: Conceptualization, Investigation, Methodology, Resources, Supervision, Writing – review & editing.

## Supplementary material

Supplementary Figure 1 Interval PET-CT scan demonstrating stable primary lesion, following pre-operative systemic therapy.

Supplementary Figure 2 Interval CT scan demonstrating radiological resolution of the previously seen peritoneal nodule, following pre-operative systemic therapy.
