# Supplementary figures and images for: Case report: From palliative to potentially curative – the advent of immunotherapy providing hope to advanced gallbladder adenocarcinoma
Source: Front Immunol. 2024 Feb 2;15:1353430. doi: 10.3389/fimmu.2024.1353430 (PMC10869450; doi:10.3389/fimmu.2024.1353430)

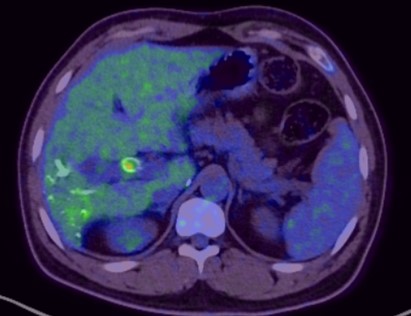

Supplement: Supplementary Figure 1 — Interval PET-CT scan demonstrating stable primary lesion, following pre-operative systemic therapy. [file Image_1.jpg]

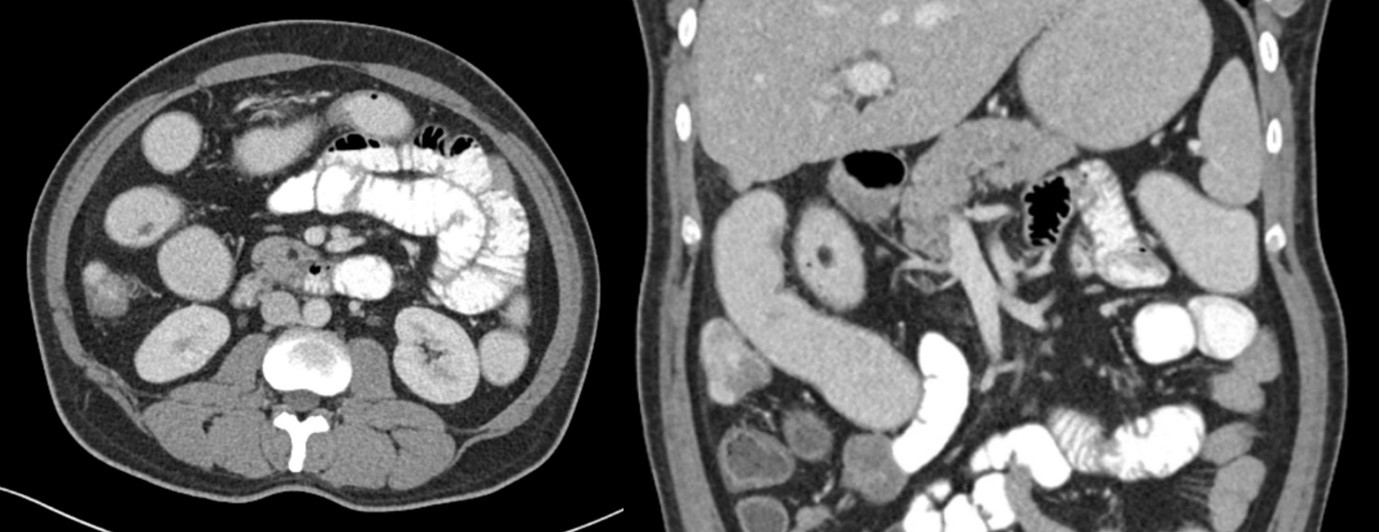

Supplement: Supplementary Figure 2 — Interval CT scan demonstrating radiological resolution of the previously seen peritoneal nodule, following pre-operative systemic therapy. [file Image_2.jpg]
